# TRIM47 is a novel endothelial activation factor that aggravates lipopolysaccharide-induced acute lung injury in mice via K63-linked ubiquitination of TRAF2

**DOI:** 10.1038/s41392-022-00953-9

**Published:** 2022-05-06

**Authors:** Yisong Qian, Ziwei Wang, Hongru Lin, Tianhua Lei, Zhou Zhou, Weilu Huang, Xuehan Wu, Li Zuo, Jie Wu, Yu Liu, Ling-Fang Wang, Xiao-Hui Guan, Ke-Yu Deng, Mingui Fu, Hong-Bo Xin

**Affiliations:** 1 The National Engineering Research Center for Bioengineering Drugs and the Technologies, Institute of Translational Medicine, Nanchang University, 1299 Xuefu Rd, Honggu District 330031 Nanchang, China; 2 Department of Biomedical Science, School of Medicine, University of Missouri Kansas City, 2411 Holmes Street, Kansas City, MO 64108 USA

**Keywords:** Infectious diseases, Inflammation

## Abstract

Endothelial activation plays an essential role in the pathogenesis of sepsis-induced acute lung injury, however, the detailed regulatory mechanisms remain largely unknown. Here, we reported that TRIM47, an E3 ubiquitin ligase of the tripartite motif-containing protein family, was highly expressed in vascular endothelial cells. TRIM47-deficient mice were effectively resistant to lipopolysaccharide (LPS)-induced acute lung injury and death by attenuating pulmonary inflammation. TRIM47 was upregulated during TNFα-induced endothelial activation in vitro. Knockdown of TRIM47 in endothelial cells inhibited the transcription of multiple pro-inflammatory cytokines, reduced monocyte adhesion and the expression of adhesion molecules, and suppressed the secretion of IL-1β and IL-6 in endothelial cells. By contrast, overexpression of TRIM47 promoted inflammatory response and monocyte adhesion upon TNFα stimulation. In addition, TRIM47 was able to activate the NF-κB and MAPK signaling pathways during endothelial activation. Furthermore, our experiments revealed that TRIM47 resulted in endothelial activation by promoting the K63-linked ubiquitination of TRAF2, a key component of the TNFα signaling pathway. Taken together, our studies demonstrated that TRIM47 as a novel activator of endothelial cells, promoted LPS-induced pulmonary inflammation and acute lung injury through potentiating the K63-linked ubiquitination of TRAF2, which in turn activates NF-κB and MAPK signaling pathways to trigger an inflammatory response in endothelial cells.

## Introduction

Acute lung injury (ALI) comprises a uniform response of the lung to inflammatory or chemical insults which is commonly caused by systemic illness including sepsis or trauma, infection with pathogens, and toxic gas inhalation.^1^ Despite a great deal of effort has been devoted to targeting the immune response to infection, there are still no effective pharmacological therapies for the treatment of ALI due to the emergence of new pathogens such as the global pandemic of novel coronavirus pneumonia, as well as the continued rise of drug resistance.^2^

As a main target of injury-related circulating cells and humoral mediators, the pulmonary endothelium is critically implicated in the pathogenesis of ALI.^1^ The interaction between endothelial cells and leukocytes is a key step in the development of ALI. Leukocyte adhesion to endothelial cells and migration across endothelial cells are mediated by the interaction of complementary adhesion molecules on leukocytes and endothelial cells. The increased expression or release of endothelial cell adhesion molecules is a hallmark of endothelial cell activation.^3,4^ Upon the access of leukocytes into the lung parenchyma, they can release inflammatory mediators to destroy pathogens, but the over-activated immune response has the potential to result in the imbalance of the pro-inflammatory and anti-inflammatory mechanisms, triggering “cytokine storm” and subsequent tissue damage.^2,5^ Considering the critical role of endothelial response during ALI, strategies that try to target endothelial components including cell surface receptors, signaling pathways, transcriptional networks, and endothelial cell gene products, have been recently proposed to attenuate endothelial activation and improve endothelial dysfunction.^6,7^

Tripartite motif-containing (TRIM) proteins, a subfamily of E3 ubiquitin ligases, are involved in many physiological processes including cell proliferation and differentiation, innate immunity, and autophagy.^8^ Multiple TRIM proteins have been found to participate in innate immunity through positive or negative regulation of cytokines, toll-like receptors, pattern recognition receptors, intracellular signaling pathways, and transcription factors.^9^ Although numerous studies have focused on the roles and regulatory mechanisms of TRIM proteins in immune cells,^10–14^ fewer studies have focused on their regulatory functions on endothelial inflammation. It has been reported that TRIM28 was abundant in endothelial cells, and interfering with TRIM28 expression has anti-inflammatory and anti-angiogenic phenotypes.^15^ However, the precise mechanism of TRIM family members in regulating endothelial activation remains largely unknown.

TRIM47 was initially found in brain astrocytomas and was named as GOA (gene overexpressed in astrocytoma).^16^ It was primarily located in the nucleus and its LXXLL motif was thought to be closely related to nuclear receptor binding.^16^ Emerging evidence indicated that TRIM47 played the roles in tumorigenesis and progression,^17–19^ viral resistance processes,^20^ and cerebral ischemia–reperfusion injury.^21^ A recent genome-wide association study showed that the SNPs of TRIM47 and TRIM65 which were located in the adjacent position of human chromosome 17, were closely related to white matter hyperintensities, a result of the ischemic damage of the small deep cerebral vessels.^22^ These findings suggested that TRIM47 and TRIM65 may coordinately or independently participate in the regulation of cerebrovascular injury. Our previous work demonstrated that TRIM65, as an E3 ubiquitin ligase, selectively promoted the ubiquitination and degradation of VCAM-1, reducing lung inflammation and damage caused by sepsis.^23^ However, the role of TRIM47 in endothelial inflammation remains to be elucidated.

In this study, a global TRIM47 knockout mouse was constructed to investigate the roles of the protein in ALI. The effects of TRIM47 on endothelial activation were also assayed in an in vitro model of inflammation induced by TNFα. Furthermore, the effects of TRIM47 on signaling pathways associated with endothelial activation were examined. Finally, we demonstrated that TRIM47 promoted pulmonary inflammation and injury through endothelial activation by potentiating the K63-linked ubiquitination of TRAF2, a key component of the TNFα signaling pathway.

## Results

### TRIM47 is highly expressed in vascular endothelial cells

**Fig. 1:**
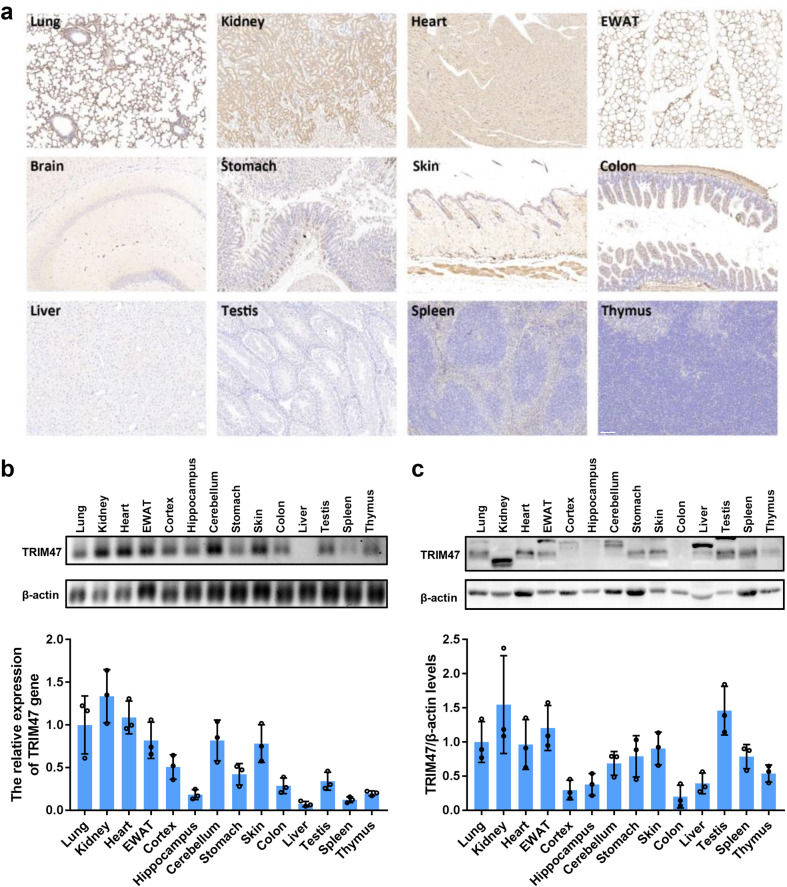
Expression of TRIM47 in tissues of mice. **a** Representative images of TRIM47 distribution in different tissues detected by immunohistochemistry (*n* = 3). Scale bar, 100 μm. **b** Representative images of agarose gel electrophoresis of TIRM47 and β-actin in various tissues of mice detected by real-time PCR (up) and the relative quantitation of TRIM47 expression (down, *n* = 3). **c** Representative images of the immunoblots of TIRM47 and β-actin in various tissues of mice detected by western blot (up) and the relative quantitation of TIRM47 protein levels (down, *n* = 3)

**Fig. 2:**
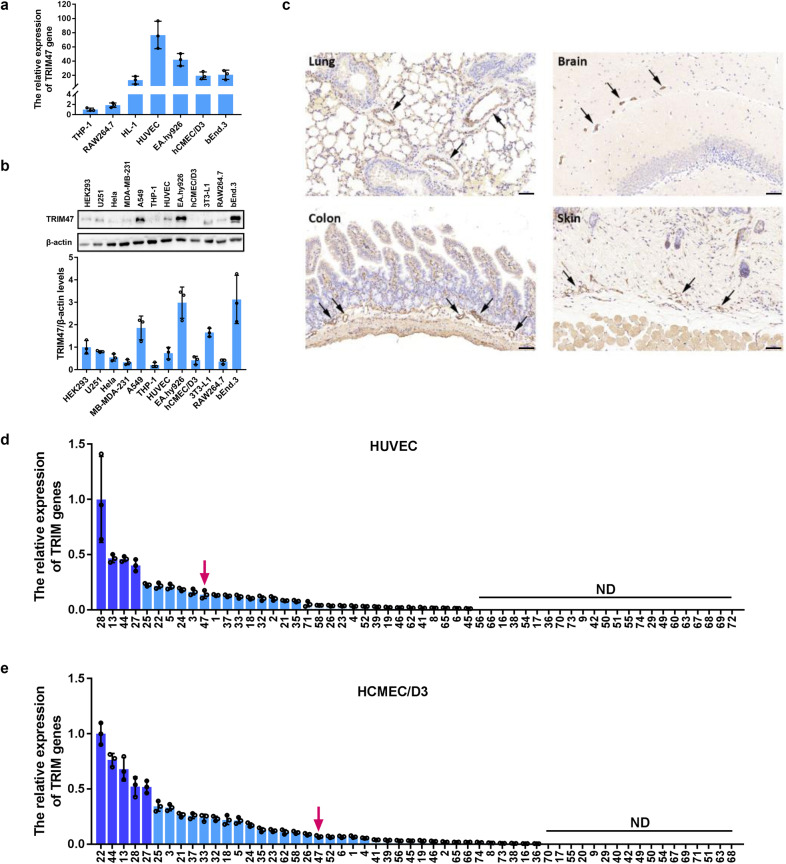
TRIM47 is highly expressed in vascular endothelial cells. **a** The relative expression of TRIM47 gene in different cells was detected by real-time PCR (*n* = 3). **b** Representative images of the immunoblots of TIRM47 and β-actin in different cells detected by western blot (up) and the relative quantitation of TIRM47 protein levels (down, *n* = 3). THP-1, human monocytic cell line, RAW264.7, murine macrophage cell line, HL-1, mouse cardiac muscle cell line, HUVEC, human umbilical vein endothelial cells, EA.hy926, human umbilical vein cell line, hCMEC/D3, human brain microvascular endothelial cell line, bEnd.3, mouse brain microvascular endothelial cell line, HEK293, Human embryonic kidney 293 cells, U251, human glioma cell line, HeLa, human cervical cancer cells, MDA-MB-231, human triple-negative breast cancer cell line, A549, human lung carcinoma cell line, and 3T3-L1, mouse preadipocytes. **c** Representative immunohistochemical images showing positive staining of TRIM47 in the vascular lining of multiple tissues (partial amplification from Fig. 1a, *n* = 3). Scale bar, 50 μm. The relative expression of 56 TRIM genes in (**d**) HUVECs and (**e**) hCMEC/D3 was detected by real-time PCR. ND: not detected (*n* = 3)

We first examined the distribution and expression of TRIM47 in various tissues of mice. As shown in Fig. 1a, the immunohistochemistry assay showed that TRIM47 had a high expression in the lung, kidney tubules, heart, and epididymal white adipose tissue (eWAT), moderate expression in the brain, stomach, skin, and colon, and low expression in the liver, testis, spleen, and thymus. Real-time PCR and western blot assay were performed to detect the mRNA and protein levels of TRIM47 in different tissues. In consistent with the immunohistochemistry results, TRIM47 mRNA was highly expressed in the lung, kidney, heart, and eWAT (Fig. 1b), and the protein was abundant in the lung, kidney, heart, eWAT, and testis (Fig. 1c). Next, we examined TRIM47 expression in a variety of cell types. Real-time PCR results revealed that TRIM47 was specifically expressed in human umbilical vein endothelial cells (HUVEC), human umbilical vein cell line EA.hy926, human brain microvascular endothelial cell line hCMEC/D3, and mouse brain microvascular endothelial cell line bEnd.3, but a low expression was observed in human monocytic cell line THP-1 and murine macrophage cell line RAW264.7 (Fig. 2a). In addition, endothelial cells exhibited high levels of TRIM47 protein detected by western blot (Fig. 2b). In particular, the immunohistochemical results showed that there was a strong expression of TRIM47 in the vascular lining of multiple tissues, as indicated by the arrows, including the lung, brain, colon, and subcutaneous tissue (Fig. 2c). Compared with the other 56 TRIM genes, TRIM47 exhibited a moderate expression in HUVECs and hCMEC/D3 respectively (Fig. 2d, e). These results indicated that TRIM47 was widely expressed in multiple tissues, and had a high expression in vascular endothelial cells.

### TRIM47 deficiency alleviates acute lung injury and inflammatory response in LPS-challenged mice

**Fig. 3:**
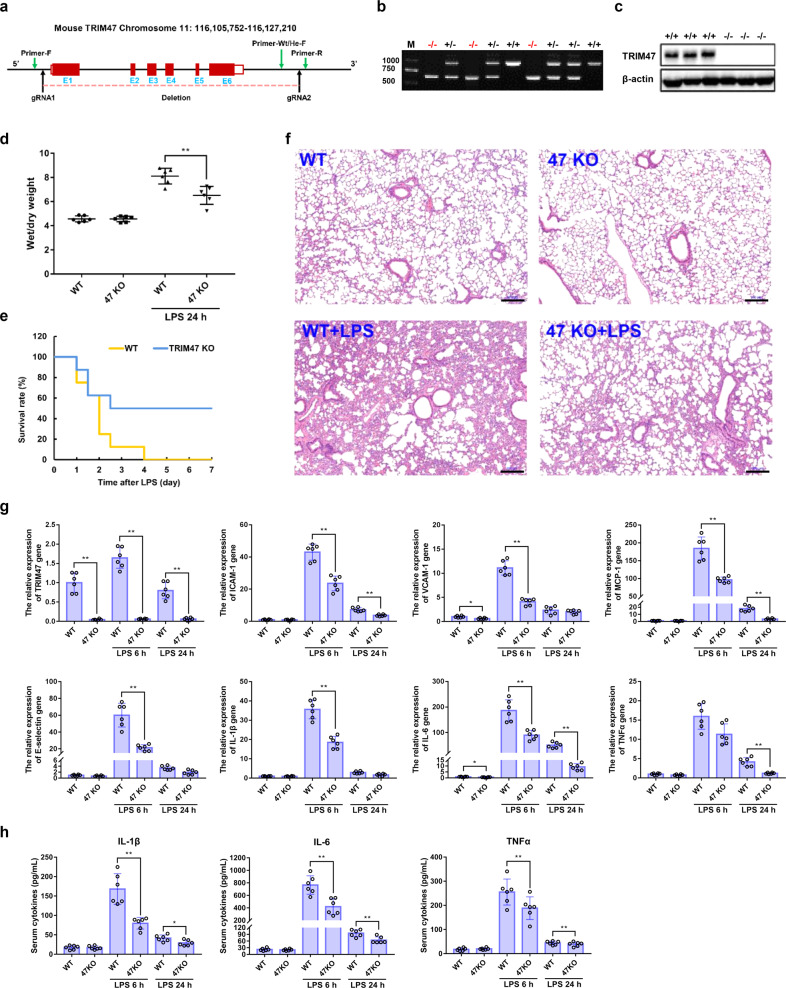
TRIM47 deficiency reduces LPS-induced acute lung injury and pulmonary inflammation in mice. **a** Schematic strategy of generation of TRIM47 knockout mice. **b** Representative images of agarose gel electrophoresis for genotyping of TRIM47^+/+^, TRIM47^+/−^, and TRIM47^−/−^ mice. **c** Representative images of the immunoblots of TIRM47 and β-actin in lungs from TRIM47^+/+^ and TRIM47^−/−^ mice detected by western blot. **d** Pulmonary edema was represented as lung wet-to-dry ratio (*n* = 6, one-way ANOVA, **P < 0.01). **e** Survival rate of mice intraperitoneally administered with 15 mg/kg LPS (*n* = 16, the log-rank test, *P* < 0.01). **f** Representative images of the histological changes in lungs after LPS challenge examined by HE staining (*n* = 6). Scale bar, 100 μm. **g** The relative expression of TRIM47 and various pro-inflammatory cytokines in lungs was measured by real-time PCR (*n* = 6, one-way ANOVA, *P < 0.05, **P < 0.01). **h** The content of IL-1β, IL-6, and TNFα in the serum was detected by ELISA (*n* = 6, one-way ANOVA, *P < 0.05, **P < 0.01)

To investigate the effects of TRIM47 on systemic inflammatory response and organ injury, a global TRIM47 knockout (TRIM47^–/−^) mouse was generated by using CRISPR/Cas9 to remove all exons of the TRIM47 gene (Fig. 3a). The knockout mice were further confirmed by RT-PCR analysis with mouse tail DNA (Fig. 3b). The lung tissue extracts were examined by western blot analysis and the results showed that TRIM47 protein was completely abolished in the homozygous targeted allele (Fig. 3c). The TRIM47^−/−^ mice had no significant changes in viscera index (Supplementary Table 1) or histology (Supplementary Fig. 1) compared with the WT mice. Lipopolysaccharide (LPS)-induced shock model was widely used to mimic the symptoms of septic shock in humans, such as systemic inflammatory responses, multiple organ dysfunction syndromes, and endotoxic shock.^24,25^ After 24 h of LPS injection, the TRIM47^−/−^ mice showed reduced pulmonary edema compared with the WT animals (Fig. 3d). In addition, TRIM47 deficiency improved the survival rate of mice after LPS challenge (Fig. 3e). HE staining showed that there were no significant differences in lung histology between WT and TRIM47^−/−^ mice under normal conditions. A significant tissue damage appeared in the lungs of WT mice, including neutrophil infiltration, alveolar wall thickening, hemorrhage, alveolar edema, and alveolar disruption (Fig. 3f). TRIM47 deficiency significantly alleviated LPS-induced histological changes in TRIM47^−/−^ mice. Next, TRIM47 and various pro-inflammatory cytokines in lungs and serum were measured after 6 h and 24 h following LPS injection, respectively. The results showed that the mRNA expression of pro-inflammatory cytokines in TRIM47^−/−^ mice were much lower than that in WT mice (Fig. 3g), and meanwhile, TRIM47 deficiency also significantly reduced LPS-induced elevations of the serum IL-1β, IL-6, and TNFα levels in mice (Fig. 3h). These results indicated that TRIM47 deficiency remarkably attenuated acute lung injury and pulmonary inflammation during LPS challenge.

### TRIM47 expression is elevated by inflammatory stimulation in endothelial cells

**Fig. 4:**
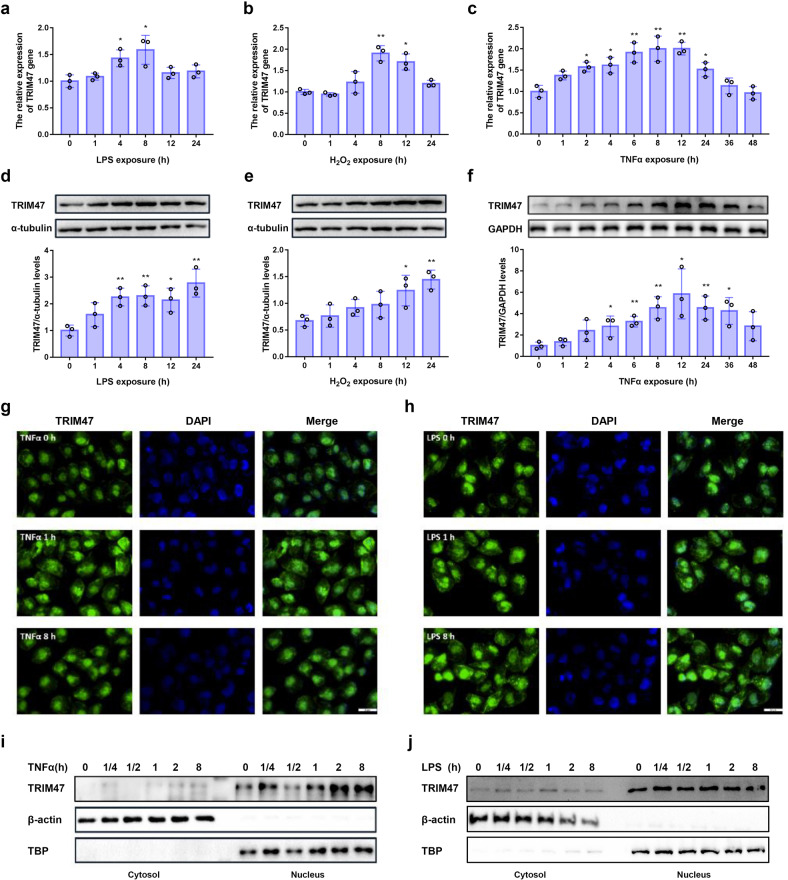
TRIM47 is induced by inflammatory stimulation in HUVECs. The expression of TRIM47 in HUVECs after (**a**) LPS, (**b**) H_2_O_2_, and (**c**) TNFα stimulation was examined by real-time PCR (*n* = 3, one-way ANOVA, *P < 0.05, **P < 0.01 compared with the 0 h group). Representative images of the immunoblots of TIRM47, α-tubulin and GAPDH in HUVECs after (**d**) LPS, (**e**) H_2_O_2_, and (**f**) TNFα stimulation detected by western blot (up) and the relative quantitation of TIRM47 protein levels (down, *n* = 3, one-way ANOVA, *P < 0.05, **P < 0.01 compared with the 0 h group). Representative images of the distribution of TRIM47 in HUVECs detected by immunocytochemistry after (**g**) TNFα and (**h**) LPS stimulation (*n* = 3). Representative images of the cytosolic and nuclear location of TRIM47 following (**i**) TNFα and (**j**) LPS treatment measured by western blot in cytosolic and nuclear fractions (*n* = 3)

The in vitro models were established to further clarify the roles of endothelial TRIM47 in inflammation and ALI. We first examined the effects of exogenous stimuli on the expression of TRIM47. Results showed that the mRNA expression of TRIM47 was significantly upregulated by LPS (Fig. 4a) and H_2_O_2_ (Fig. 4b). The expression of TRIM47 was significantly increased since 2 h after TNFα stimulation and peaked at 12 h (Fig. 4c). TRIM47 protein levels were also upregulated in response to inflammation or oxidative stress (Fig. 4d–f). The immunocytochemistry assay confirmed that TRIM47 mainly located in the nucleus of HUVECs. An increased expression of TRIM47 was found in both cytosol and nucleus after 1 h of TNFα stimulation and the increase lasted for 8 h (Fig. 4g). LPS induced an increase in cytosolic TRIM47 fluorescence at both 1-h and 8-h time points (Fig. 4h). In addition, the localization of TRIM47 was determined by western blot in the cytosolic and nuclear fractions, respectively. The results showed that the nucleus contains a prominent proportion of TRIM47, which was significantly upregulated upon TNFα stimulation. The cytosolic TRIM47 contents were also increased after TNFα stimulation (Fig. 4i). LPS induced a slight increase in cytosolic TRIM47, which was not changed significantly in the nucleus (Fig. 4j). Considering the heterogeneity of endothelial cells, the expression profile of TRIM47 was also determined in endothelial cells from different vessels. TRIM47 expression was also upregulated in bEnd.3 cell line upon various stimulation, including inflammation and hypoxia (Supplementary Fig. 2a–d). The immunostaining showed stronger fluorescence intensity of TRIM47 expression in hCMEC/D3 cell line following TNFα and LPS incubation (Supplementary Fig. 2i, j). Since macrophages are activated during the inflammatory process, we also evaluated the alteration of TRIM47 expression in macrophages. TRIM47 was not affected by TNFα challenge but remarkably decreased by LPS exposure (Supplementary Fig. 2e–h). Taken together, TRIM47 expression is elevated by inflammatory stimulation and might play a role in the inflammatory response of endothelial cells. Since TNFα is the strongest stimuli in triggering and amplifying endothelial inflammatory response due to the TNF-specific membrane receptors, TNFR1 and TNFR2,^26^ and TRIM47 expression is much more sensitive to TNFα stimulation, TNFα was used to induce endothelial inflammation in the following study.

### TRIM47 promotes TNFα-induced endothelial activation

**Fig. 5:**
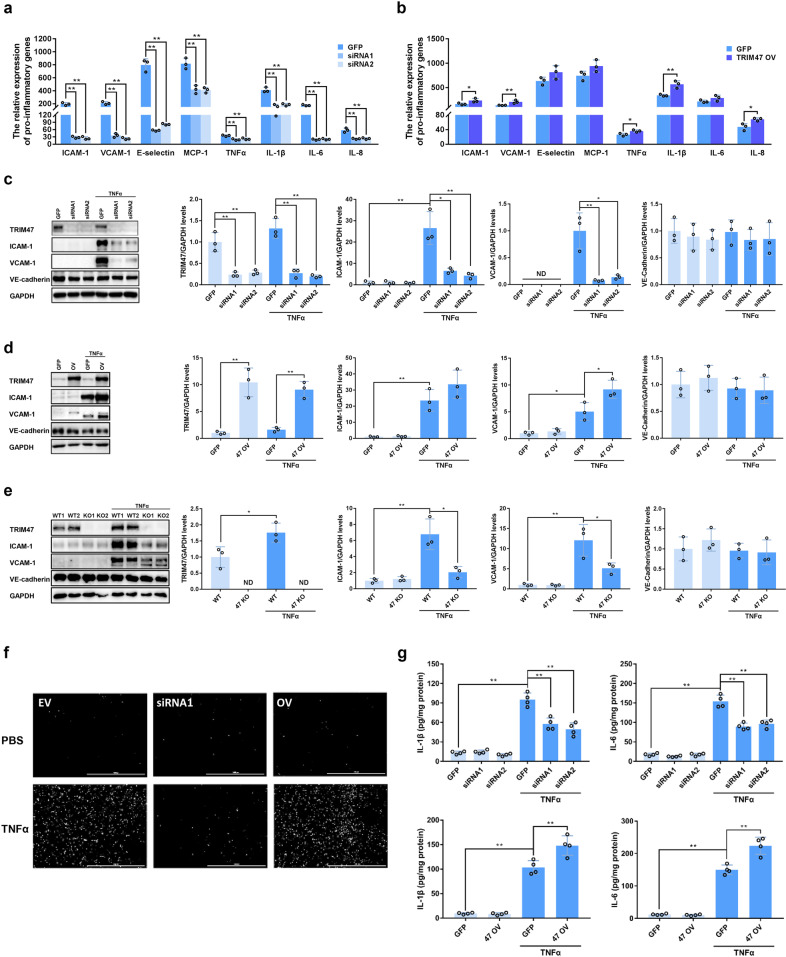
TRIM47 promotes inflammatory response in endothelial cells. The mRNA expression of multiple adhesion molecules and pro-inflammatory cytokines was detected by real-time PCR in (**a**) TRIM47 siRNA- and (**b**) overexpression vector-transfected HUVECs (*n* = 3, one-way ANOVA, *P < 0.05, **P < 0.01). Representative images of the immunoblots of TIRM47, ICAM-1, VCAM-1, VE-cadherin, and GAPDH after TNFα stimulation detected by western blot and the relative quantitation of the protein levels in (**c**) TRIM47 knockdown HUVECs, (**d**) TRIM47 overexpression HUVECs, and (**e**) the pulmonary microvascular endothelial cells from wild-type (WT) and TRIM47 Knockout (KO) mice. ND not detected. (*n* = 3, one-way ANOVA, *P < 0.05, **P < 0.01). **f** Representative images of the adhesion of THP-1 to siRNA- and overexpression vector-transfected HUVECs were observed under a fluorescence microscope (*n* = 3). Scale bar, 1000 μm. **g** The levels of IL-1β and IL-6 in siRNA- and overexpression vector-transfected cells were measured by ELISA (*n* = 4, one-way ANOVA, **P < 0.01)

The siRNA and overexpression vectors were constructed to explore the role of TRIM47 in TNFα-induced endothelial activation. Knockdown of TIRM47 significantly reduced TNFα-induced mRNA expression of multiple adhesion molecules such as ICAM-1, VCAM-1, E-selectin, and MCP-1, and pro-inflammatory cytokines such as TNFα, IL-1β, IL-6, and IL-8 in HUVECs (Fig. 5a). By contrast, overexpression of TRIM47 remarkably enhanced the mRNA levels of these molecules (Fig. 5b). Western blot results showed that knockdown of TRIM47 markedly inhibited the expression of ICAM-1 and VCAM-1 induced by TNFα (Fig. 5c), whereas TRIM47 overexpression promoted the expression of these proteins (Fig. 5d). The effects of TRIM47 on adhesion molecules expression were further confirmed in pulmonary microvascular endothelial cells from the wild-type and TRIM47 KO mice. As expected, TNFα-induced elevations of ICAM-1 and VCAM-1 were significantly suppressed in TRIM47-deficient pulmonary microvascular endothelial cells (Fig. 5e). Furthermore, knockdown of TRIM47 significantly reduced TNFα-induced adhesion of THP-1 cells to HUVECs, but overexpression of TRIM47 had the opposite effects (Fig. 5f). TRIM47 knockdown also suppressed TNFα-induced secretion of pro-inflammatory cytokines such as IL-1β and IL-6. By contrast, overexpression of TRIM47 promoted TNFα-induced production of pro-inflammatory cytokines (Fig. 5g). In addition, knockdown of TRIM47 remarkably inhibited cell proliferation and migration induced by TNFα in HUVECs (Supplementary Fig. 3). These results indicated that TRIM47 was a positive regulator of TNFα-induced endothelial activation.

### TRIM47 activates NF-κB and MAPK pro-inflammatory signaling pathways

**Fig. 6:**
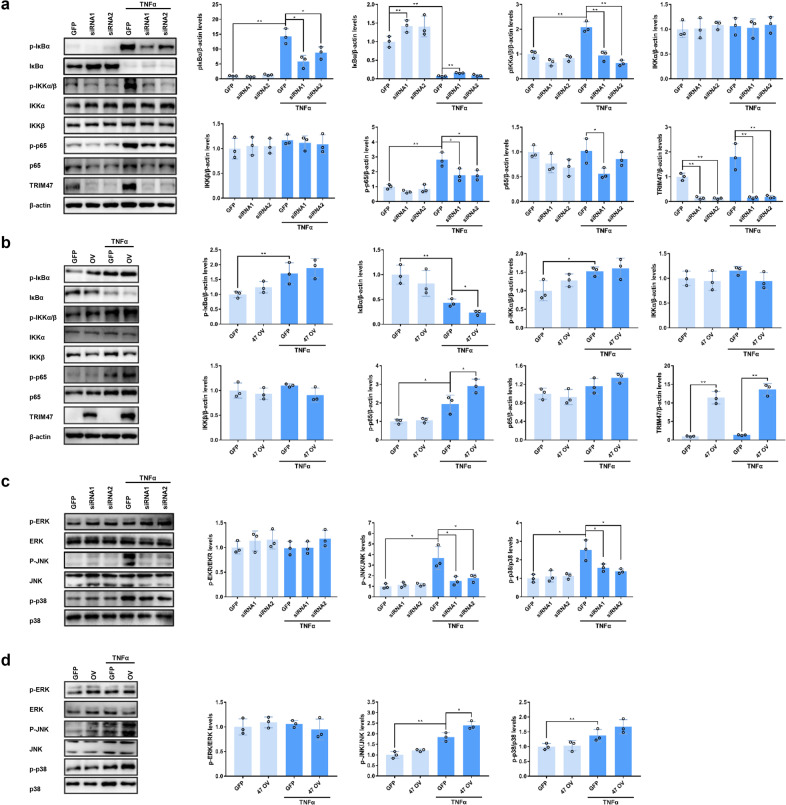
TRIM47 modulates endothelial activation through NF-κB and MAPK signaling pathways. Representative images of the immunoblots of p-IκBα, IκBα, p-IKKα/β, IKKα, IKKβ, p-p65, p65, TIRM47, and β-actin after TNFα stimulation detected by western blot and the relative quantitation of the protein levels in (**a**) TRIM47-knockdown and (**b**) TRIM47-overexpressed HUVECs. Representative images of the immunoblots of p-ERK, ERK, p-JNK, JNK, p-p38, and p38 after TNFα stimulation detected by western blot and the relative quantitation of the protein levels in (**c**) TRIM47-knockdown and (**d**) TRIM47-overexpressed HUVECs (*n* = 3, one-way ANOVA, *P < 0.05, **P < 0.01)

The potential signaling pathways including NF-κB and MAPK in TRIM47-mediated endothelial activation were investigated. The results showed that knockdown of TRIM47 significantly inhibited the phosphorylation of IκBα, IKKα/β, and p65 subunit, and prevented the degradation of IκBα (Fig. 6a), whereas overexpression of TRIM47 promoted the activation of the NF-κB signaling pathway (Fig. 6b). TRIM47 knockdown suppressed the activation of JNK and p38 MAPK signal pathways but had no obvious effect on ERK (Fig. 6c). TRIM47 overexpression further activated JNK and p38 signal pathways after TNFα stimulation (Fig. 6d). These results indicated that TRIM47-mediated endothelial activation might be related to the activation of NF-κB and MAPK signaling pathways.

### TRIM47 promotes K63-linked ubiquitylation of TRAF2

**Fig. 7:**
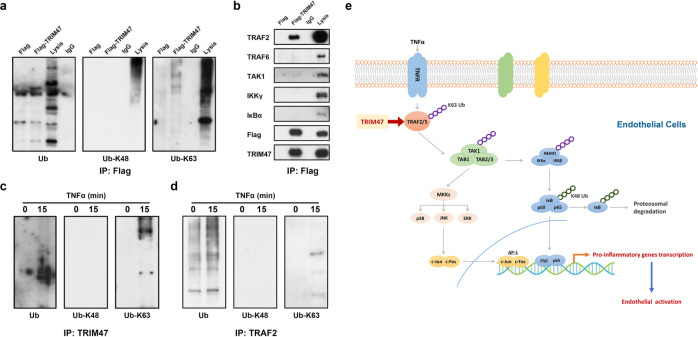
TRIM47 mediates K63-linked ubiquitylation and interacts with TRAF2. HUVECs were transfected with Flag or Flag-TRIM47 plasmid for 48 h and treated with TNFα for 15 min. Whole-cell lysates were immunoprecipitated with Flag antibody, and the precipitates were immunoblotted with (**a**) Ub, Ub-K48, Ub-K63, (**b**) TRAF2, TRAF6, TAK1, IKKγ, and IκBα antibodies (*n* = 3). HUVECs were treated with 10 ng/ml TNF-α for 0 and 15 min. Cell lysates were then immunoprecipitated using (**c**) TRIM47 and (**d**) TRAF2 antibodies. Representative images demonstrated the total, K48- and K63-linked ubiquitylation by western blot analysis using the indicated antibodies (*n* = 3). **e** The schematic diagram of the mechanism by which TRIM47 mediates TNFα-induced endothelial activation through promoting endothelial TRAF2-MAPK/NF-κB pro-inflammatory axis

Emerging evidence showed that TRIM proteins mediated K48- or K63-linked ubiquitination to activate NF-κB signaling pathway in response to exogenous stimulation.^12,27^ Therefore, the ubiquitination pattern of TRIM47 involved in endothelial activation was analyzed. The Flag or Flag-TRIM47 plasmid was transfected into HUVECs for 48 h. The transfected cultures were treated with 10 ng/mL TNFα for 15 min before harvesting. The whole-cell lysates were immunoprecipitated with the Flag antibody and the complex was detected with ubiquitin antibodies. Overexpression of TRIM47 obviously enhanced K63-linked ubiquitination rather than K48 (Fig. 7a). Tumor necrosis factor receptor-associated factor 2 (TRAF2) is a key adaptor molecule in TNFR signaling complexes that promotes downstream signaling cascades, such as NF-κB and MAPK activation,^28^ whereas TRAF6 is the major transducer of IL-1 receptor/TLR signaling.^29^ It has been reported that the K63-linked polyubiquitin chains could be attached to TRAF2, serving as a scaffold to recruit TAK1, TAB1, and TAB2. The active TAK1 further phosphorylates the MAPKs and IKK complex to initiate MAPK and NF-κB cascades.^30^ We, therefore, examined the possible binding proteins of TRIM47 involved in this signal pathway. As shown in Fig. 7b, TRIM47 bound with TRAF2 but not TRAF6. TRIM47 did not interact with the downstream proteins, including TAK1, IKKγ, and IκBα. In addition, TRIM47 did not induce the degradation of IκBα, a classical target of K48-linked ubiquitination (Supplementary Fig. 4). The co-immunoprecipitation assay with TRIM47 and TRAF2 antibodies further confirmed that endogenous TRIM47 and TFAF2 were involved in K63-linked ubiquitination in HUVECs (Fig. 7c, d). These results indicated that TRIM47 regulated NF-κB and MAPK activation possibly by enhancing the K63-linked ubiquitination of TRAF2.

## Discussion

In this study, we observed that TRIM47 deficiency significantly alleviated LPS-induced pulmonary inflammation and tissue damage in mice. We further demonstrated that TRIM47 was an activator of TNFα-induced endothelial activation. As a potential E3 ubiquitin ligase, TRIM47 interacts with TRAF2 and mediates K63-linked ubiquitination, thus activating NF-κB and MAPK signaling pathways. Obviously, our results provided strong evidence that TRIM47-mediated acute lung injury was related with its induction in endothelial activation.

TRIM47 was initially found to be highly expressed in astrocytoma tumor cells and astrocytes of the fetal brain, with prominent nuclear staining, but absence in mature astrocytes. The expression of TRIM47 in normal tissues was low except for in the kidney.^16^ Here, we showed that TRIM47 has relatively high expression in the heart, lung, kidney, and eWAT by the immunohistochemistry assay and detection of mRNA and protein levels in these tissues. Interestingly, the vascular endothelial cells from different tissues exhibited remarkable positive staining of TRIM47. The samples from cell cultures further confirmed that its prominent expression in various endothelial cells. These results suggested that TRIM47 might serve as a vascular endothelial-specific protein and regulate endothelial functions.

Many TRIM proteins have been proved to be implicated in innate immunity and inflammatory response. Our previous work showed that TRIM47 was down-regulated upon TNFα exposure in THP-1-derived macrophages.^31^ In the present study, we showed that TRIM47 was significantly induced by multiple stimuli, including TNFα, LPS, hypoxia, and oxidative stress in endothelial cells, indicating that TRIM47 might be a sensor in response to exogenous stimulation. Consistent with the previous report,^16^ we observed that TRIM47 was prominently located in the nucleus of HUVECs. Both nuclear and cytosolic TRIM47 were upregulated after stimulation, but there were no evidence showing the translocation of TRIM47. In addition, no typical nuclear localization sequence (NLS) of TRIM47 was found by cNLS Mapper or NLStradamus Software. Whether TRIM47-mediated endothelial activation depends on its subcellular location needs further investigation in the future.

Different from the anti-inflammatory effect of TRIM65, another constitutively expressed protein in endothelial cells,^23^ TRIM47 promotes inflammatory response both in vitro and in vivo. TRIM47 knockdown showed strong inhibition of adhesion molecules expression and monocyte adhesion in HUVECs. These effects were further confirmed in pulmonary microvascular endothelial cells from TRIM47 KO mice, indicating that TRIM47-mediated endothelial activation mainly contributed to the pathological process of acute lung injury. Since the previous GWAS results suggested that both TRIM47 and TRIM65 are closely related to the cerebral vessels injury,^22^ our results provided strong evidence that although TRIM47 and TRIM65 were critically involved in the regulation of endothelial inflammation and injury, their mechanisms might differ.

Proteins covalently modified with K48-linked polyubiquitin are targeted for proteasomal degradation, whereas proteins covalently modified with K63-linked polyubiquitin generally become functionally activated.^9^ Recent work showed that TRIM proteins positively or negatively mediated NF-κB activation through K48- or K63-linked polyubiquitin, respectively. For example, TRIM5 interacted with the TAK1-containing kinase complex to positively regulate NF-κB activation by mediating K63 polyubiquitin chain synthesis.^12^ TRIM25 promoted NF-κB activation by enhancing the K63-linked ubiquitination of TRAF2 and bridging the interaction of TRAF2 and TAK1 or IKKβ.^27^ TRIM14 enhanced NF-κB activation in endothelial cells via directly binding to NEMO and promoting the phosphorylation of IκBα and p65, which was dependent on its K63-linked ubiquitination.^32^ Several target proteins of TRIM47 have been found in recent years. For example, TRIM47 induced ubiquitination of PPM1A and decreased the expression level of PPM1A in human embryonic lung fibroblast.^33^ TRIM47 interacted with CYLD and induced its degradation in proportion to non-alcoholic steatohepatitis severity.^34^ As an oncogene, TRIM47 promoted cancer proliferation through ubiquitination and degradation of multiple targets such as FOXO1,^35^ P53,^36^ SMAD4^18^ and FBP1.^37^ TRIM47 was also implicated in innate immunity through K48-linked ubiquitination of NF90.^38^ Structurally similar to TRIM25, a reported E3 ligase facilitating K63-linked ubiquitination,^8^ TRIM47 also interacts with TRAF2 and promotes MAPK and NF-κB activation through K63-linked ubiquitination. Taken together, we provided evidence that E3 ligase TRIM47 aggravated TNFα-induced endothelial activation and acute lung injury through selectively promoting the K63-linked ubiquitination of TRAF2, a key component of the TNFα signaling pathway.

To sum up, we identified TRIM47 is a novel activator of endothelial cells, in which it mediates inflammatory response and promotes inflammation and tissue damage during ALI through activating endothelial TRAF2-MAPK/NF-κB pro-inflammatory axis (Fig. 7e). At present, no compounds that targeting TRIM proteins have been identified, but it is important to develop the specific inhibitors of TRIM proteins as the therapeutic tools in multiple diseases.^8^ TRIM47 may be an attractive target for drug development in endothelial inflammation and ALI. Certainly, further elucidating the underlying mechanism of TRIM47 in endothelial activation should provide an insight into the development of effective therapeutic agents clinically.

## Materials and methods

### Reagents

Recombinant Human and murine TNFα were obtained from Peprotech. LPS (from *Escherichia coli* O111:B4) was purchased from Sigma. VCAM-1 (sc-13160), ICAM-1 (sc-1511-R) and β-actin (sc-47778) antibodies were from Santa Cruz Biotechnology. TRIM47 antibody (26885-1-AP) was purchased from Proteintech. Phospho-p65 (3033), p65 (8242), IκBα (4812), phospho-IκBα (2859), phospho-IKKα/β (2078), IKKα (11930), IKKβ (8943), phospho-JNK (4668), JNK (9252), phospho-ERK1/2 (4370), ERK1/2 (4695), phospho-p38 (4511), p38 (8690),TRAF2 (4724), TRAF6 (8028), TAK1(5206), IKKγ (2685), TBP (44059), Flag (8146 and 2368) and α-tubulin (2125) antibodies were purchased from Cell Signaling Technology. VE-cadherin (ab33168), ubiquitin (ab7780), ubiquitin (K48, ab140601), and ubiquitin (K63, ab179434) antibodies were purchased from Abcam.

### Generation of TRIM47 knockout mice

All procedures related to the care of animals were performed according to the National Institutes of Health Guide for the Care and Use of Laboratory Animals. All experimental protocols were approved by Institutional Animal Care and use Committee of Nanchang University. To define the physiological role of TRIM47 in vivo, we have obtained the mice with heterozygous TRIM47-targeted allele by using CRISPR/Cas9 to remove all exons of TRIM47 gene. The mice with homozygous TRIM47-targeted alleles were generated by interbreeding. The mice were created in C57BL/6J background. Genotyping was done with the following primers: Trim47-F: 5′-GGTAAACACAGTCGCTAAGAGGTCAAA-3′, Trim47-R: 5′-TGGTCTAGGGATGCCAGGGTTCT-3′, and Trim47-Wt/He-F: 5′-AGTCAGAGTGAGCAGGCAGGAGAATA-3′. Wild-type and TRIM47 knockout mice were housed in the Animal Centre of Institute of Translational Medicine, Nanchang University, with a 12 h light–dark cycle, optimal temperature and humidity, filtered water, and appropriate nutrient feed.

### LPS challenge in mice

Age-matched mice (7–9 weeks) were randomly assigned to control or experimental groups. Wild-type and TRIM47^−/−^ mice underwent an intraperitoneal injection of LPS (15 mg/kg) to induce lethal endotoxic shock. The control group received injections of the equivalent volume of 0.9% NaCl solution. After injection, the mice were closely monitored for general condition and survival for 7 days.

### Histological analysis

The right upper lungs were removed after 24 h of LPS challenge and were fixed in 4% phosphate-buffered paraformaldehyde. The 4-μm paraffin tissue sections were cut and stained with H&E as previously described.^39^ Photomicrographs were taken by a light microscope (Olympus BX51). Lung injury was evaluated by an independent pathologist who was blinded to the grouping, including hemorrhage in the lung tissue, alveolar congestion, edema, infiltration of macrophages and neutrophils, and morphological changes in the alveolar wall. For immunostaining, the paraffin tissue sections were incubated in 3% H_2_O_2_ to inactivate endogenous peroxidases. Antigen retrieval was performed by heating the sections in citrate buffer. Endogenous biotin was blocked with the Biotin-Blocking Kit (Maixin Biotechnologies, Fuzhou, China). Sections were then incubated with the anti-TRIM47 antibody for 60 min at a 1:200 dilution, followed by HRP-conjugated secondary antibodies for 30 min. Detection was performed using DAB substrate kit (Maixin Biotechnologies).

### Measurement of lung wet/dry weight ratio

To evaluate the magnitude of pulmonary edema, the wet-to-dry weight ratios at 24 h after LPS challenge were determined. The left lung tissue samples were weighed immediately after removal (wet weight) and then subjected to desiccation in an oven at 50 °C until a stable dry weight was achieved after 72 h. The ratio of the wet/dry weight was then calculated.

### Cell culture, infection, and treatment

Human Umbilical Vein Endothelial Cells (HUVEC) and THP-1 cells were purchased from Lonza Walkersville Inc. HUVECs were cultured in EGM medium according to the manufacturer’s instruction, and used for the experiment in less than eight passages. THP-1 was cultured in RPMI-1640 medium supplemented with 10% FBS and 2-mercaptoethanol to a final concentration of 0.05 mM. RAW264.7, EA.hy926, bEnd.3, and HEK293 cells were purchased from ATCC and cultured in DMEM supplemented with 10% FBS. U251, HeLa, MDA-MB-231, 3T3-L1, and A549 cell lines were from National Collection of Authenticated Cell Cultures (Shanghai, China) and cultured in DMEM supplemented with 10% FBS. The hCMEC/D3 cell line was purchased from BeNa Culture Collection (Beijing, China) and cultured in RPMI-1640 medium supplemented with 10% FBS. HL-1 cardiac muscle cell line was obtained from Sigma-Aldrich and cultured in Claycomb medium supplemented with 100 μM norepinephrine, 4 mM l-glutamine and 10% FBS. Two siRNA target sequences were selected in Human TRIM47 gene (GenBank accession NM_033452.2): siRNA1: 5′-TGAAGCTCCCAGGGACTATTT-3′, and siRNA2: 5′-TACTGGGAGGTGGAGATTATC-3′. TRIM47 siRNA was constructed into the lentivirus expression vector pLV[shRNA]-EGFP:T2A:Puro-U6. A universal sequence was used as a negative control for RNA interference. Human TRIM47 gene was constructed into pLV[Exp]-EGFP:T2A:Puro-EF1A vector to obtain the expression lentiviral vector. The viral particles were produced by third-generation packaging in 293T cells and Lentiviral stocks were concentrated using ultracentrifugation. HUVECs (5 × 10^4^/ml) were prepared and infected at a Multiplicity of Infection (MOI) of 50 with negative control, TRIM47 siRNA1, TRIM47 siRNA2 or TRIM47 overexpression lentiviruses for 16 h at 37 °C in the presence of 5 μg/ml polybrene. The cultures were then washed and cultured in fresh medium for 72 h. GFP expression was detected to calculate the infection efficiency. Then, cells were treated with 10 ng/ml TNFα for indicated times, and mRNAs or proteins from those cells were extracted and detected.

### Pulmonary microvascular endothelial cell isolation

The pulmonary microvascular endothelial cells were isolated from wild-type and TRIM47 KO mice, respectively, according to the previously described method.^23^ Briefly, the mice were euthanized under deep isoflurane anesthesia, and lungs were rapidly removed on ice. Lung tissue was gently homogenized and digested by collagenase and DNAase for 1 h at 37 °C. The cells were filtered through a 70-μm cell strainer, washed with PBS containing 2 mM EDTA and 0.5% BSA, and collected by centrifugation. The endothelial cells were isolated by MACS magnetic cell sorting (Milteryi Biotec) with positive selection by the CD31 MicroBeads (130-097-418, Milteryi Biotec) according to the manufacturer’s instructions. Enriched primary endothelial cells were incubated with 10 μl of mouse CD31 Microbeads at 4 °C for 15 min and were centrifuged at 300×*g* for 10 min. CD31-positive cells were collected on a MACS LS column and washed three times with PBS containing 10% FBS. The cells were re-suspended and cultured in endothelial cell basal medium-2 (EBM-2, Lonza). The pulmonary microvascular endothelial cells were treated with 10 ng/ml murine TNFα for 8 h. The protein levels of ICAM-1 and VCAM-1 were detected by western blot.

### RNA isolation and qPCR

Total tissue or cellular RNA was isolated using TRIzol reagent, according to the manufacturer’s instructions (Life Technologies, CA, USA). One microgram of total RNA was reverse-transcribed using a One Step PrimeScript™ RT-PCR Kit (Takara, Liaoning, China) with a thermocycler. The mRNA levels were determined by SYBR Green dye using an ABI 7500 sequence detection system with a reaction mixture that consisted of SYBR Green 2×PCR Master Mix (Applied Biosystems, CA, USA), cDNA template, and the forward and reverse primers. Primer sequences were listed in Supplementary Tab. 2. The PCR protocol consisted of 40 cycles of denaturation at 95 °C for 15 s followed by 60 °C for 1 min to allow extension and amplification of the target sequence. Data were analyzed using ABI 7500 sequence detection system software. The amount of mRNA was normalized to GAPDH using the 2^−ΔΔCT^ method. The results were from three independent experiments performed in triplicate.

### Protein isolation and western blot

Tissue extracts and whole-cell lysates were prepared in radioimmunoprecipitation assay buffer (Thermo Scientific) supplemented with 1 mM PMSF. Nuclear and cytoplasmic protein fractions from cells were extracted by Nuclear-Cytosol Extraction Kit (Applygen Technologies Inc, Beijing, China), according to the manufacturer’s instructions. Fifty micrograms protein per sample was loaded in each lane and separated by sodium dodecyl sulfate-polyacrylamide gel electrophoresis (SDS-PAGE) and transferred to nitrocellulose membranes (Pall Corporation, USA) in Tris-glycine buffer (48 mM Tris, 39 mM glycine, pH 9.2) containing 20% methanol. The membranes were blocked with skimmed milk for 1 h, washed in Tris-buffered saline containing 0.1% Tween-20 (TBST), and incubated with primary antibodies overnight at 4 °C. After washing in TBST for three times, nitrocellulose membranes were incubated for 1 h at room temperature with the horseradish peroxidase-conjugated IgG (1:5000; Santa Cruz Biotechnology, Inc, CA, USA). The bands were visualized by the SuperSignal West Pico Chemiluminescent Substrate Trial Kit (Pierce, Rockford, IL, USA). The immunodetected protein bands were then analyzed using ChemiDoc XRS system with Quantity One software (Bio-Rad, Richmond, CA, USA).

### Immunocytochemistry

At the end of the treatment, cells were rinsed with phosphate-buffered saline (PBS) three times, fixed with 4% paraformaldehyde for 30 min at room temperature, and permeabilized in 0.1% Triton X-100 for 10 min. An incubation in 5% bovine serum albumin (BSA) in PBS for 1 h was performed to prevent antibody non-specific binding. The cultures were incubated with primary antibodies overnight at 4 °C. After incubation with primary antibodies, cells were incubated with fluorescein isothiocyanate (FITC)-conjugated goat anti-rabbit IgG (Alexa 488; 1:1000; Invitrogen), and the nuclei were stained with DAPI. Immunostained cells were examined under a fluorescence microscope (Olympus IX71, Tokyo, Japan).

### Monocyte adhesion assay

The adhesion assay was performed as previously described.^23^ The control, TRIM47 knockdown, or TRIM47 overexpressed HUVECs were cultured on a six-well plate overnight, and then were treated with 10 ng/ml TNF-α for 8 h. THP-1 cells were labeled with a Zombie Red fluorescent staining kit (Biolegend, San Diego, CA) according to the manufacturer’s instructions. The labeled THP-1 cells were added into HUVECs at a density of 5 × 10^5^ per well and were co-cultured for 1 h at 37 °C. Non-adherent cells were removed by gently washing with cold RPMI-1640 medium. The images of adherent THP-1 cells and the number were determined under Cytation 3 Cell Imaging Multi-mode Reader (Biotek Instruments).

### Measurements of inflammatory cytokines

The concentrations of TNFα, IL-1β, and IL-6 in the serum, and IL-1β and IL-6 levels in the supernatant of HUVECs were measured using the specific ELISA kits according to the manufacturer’s instructions (Neobioscience Technology Co., Ltd., Shenzhen, China). Absorbance at 450 nm wavelength was measured, and the protein concentration was determined by interpolation on to absorbance curves generated by recombinant protein standards using iMark™ Microplate Absorbance Reader (Bio-Rad).

### Ubiquitination assay with Co-IP

HUVECs were transfected with Flag or Flag-TRIM47 plasmid using electroporation. The protease inhibitor MG132 (10 μM; Sigma-Aldrich) was added for 1 h before harvesting. After 48 h of transfection, cells were lysed in CelLytic M Cell lysis buffer with protease inhibitors, phosphatase inhibitors, NEM, and ubiquitin aldehyde. HUVECs were harvested after 15 min-TNFα (10 ng/mL) exposure. The immune complexes were collected by incubation (2 h, 4 °C) with protein G-agarose (Sigma). Co-IP assays were performed by using Pierce^TM^ Protein G-Agarose (Thermo Fisher) following the manufacturer’s instructions. After extensive washing, the electrophoresis loading buffer was added to the complexes and incubated for 5 min at 95 °C. Immunoprecipitated proteins were resolved by SDS-PAGE and analyzed by immunoblotting with indicated antibodies.

### Statistics

Statistical analysis was performed with GraphPad Prism software (GraphPad, San Diego, CA). Data were expressed as mean ± SD. For comparison between two groups, the unpaired Student’s *t* test was used. For multiple comparisons, one-way ANOVA followed by Turkey’s post hoc analysis was used. The survival rate comparisons were made by the log-rank test. A value of *P* < 0.05 was considered significant.

## Supplementary information

Supplementary Materials

## Data Availability

All data that support the findings of this study are available from the corresponding authors upon reasonable request.
