# Supplementary material for: TRIM47 is a novel endothelial activation factor that aggravates lipopolysaccharide-induced acute lung injury in mice via K63-linked ubiquitination of TRAF2
Source: Signal Transduct Target Ther. 2022 May 6;7:148. doi: 10.1038/s41392-022-00953-9 (PMC9072678; doi:10.1038/s41392-022-00953-9)
Supplement: Supplementary file 1 — Supplementary Materials [file 41392_2022_953_MOESM1_ESM.docx]

Supplementary Materials for

TRIM47 is a novel endothelial activation factor that aggravates lipopolysaccharide-induced acute lung injury in mice via K63-linked ubiquitination of TRAF2

Yisong Qian, Ziwei Wang, Hongru Lin, Tianhua Lei, Zhou Zhou, Weilu Huang, Xuehan Wu, Li Zuo, Jie Wu, Yu Liu, Ling-Fang Wang, Xiao-Hui Guan, Ke-Yu Deng, Mingui Fu^2^, Hong-Bo Xin^*^

Correspondence to: xinhb@ncu.edu.cn or fum@umkc.edu.

**This PDF file includes:**

Figures. S1 to S4

Tables S1 to S2


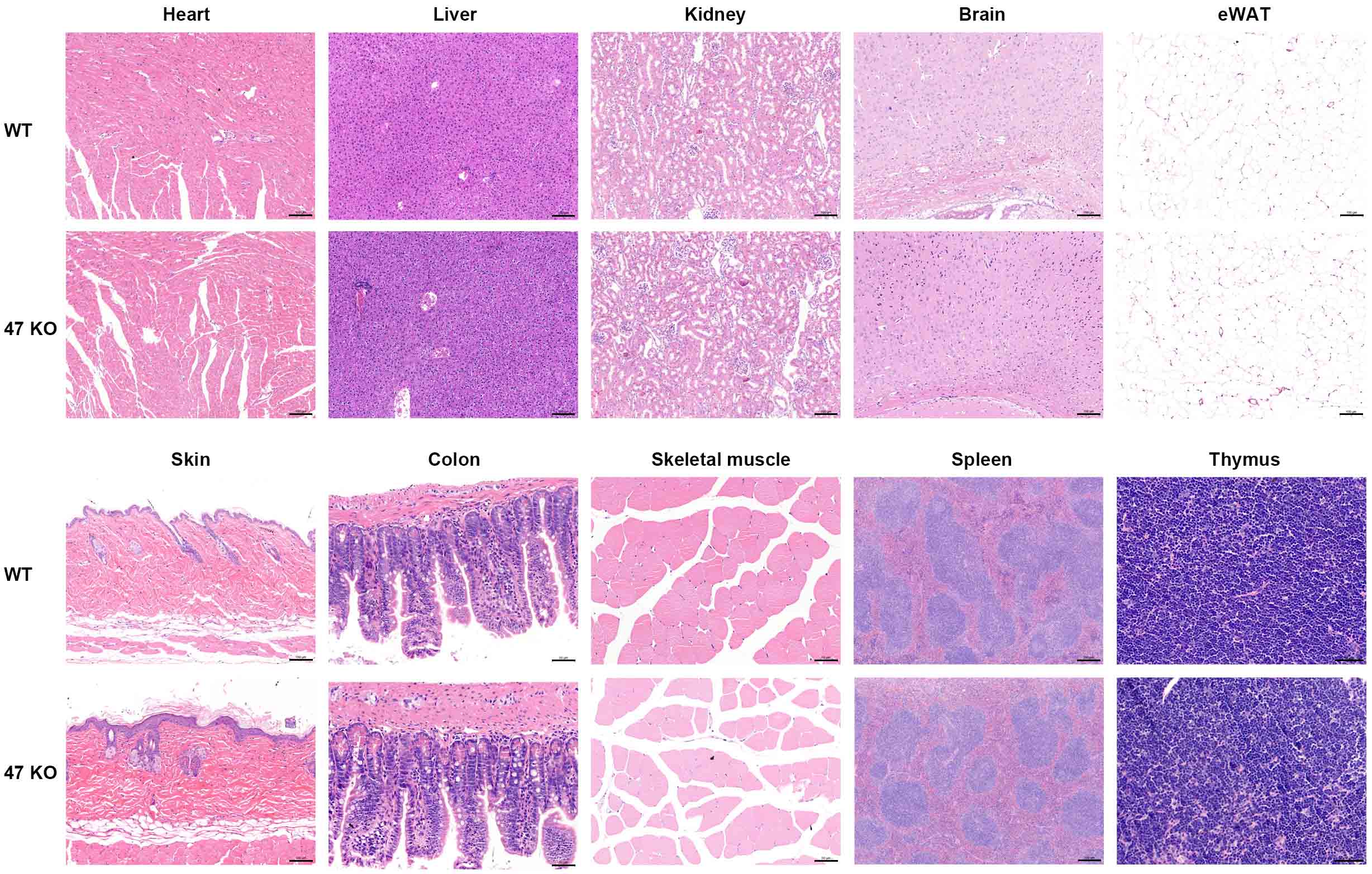


Figure. S1. Representative images of histology measurement by HE staining in wild type (WT) and TRIM47 knockout (47 KO) mice. No obvious changes were observed in TRIM47 deficient mice compared with the WT mice (n = 6).


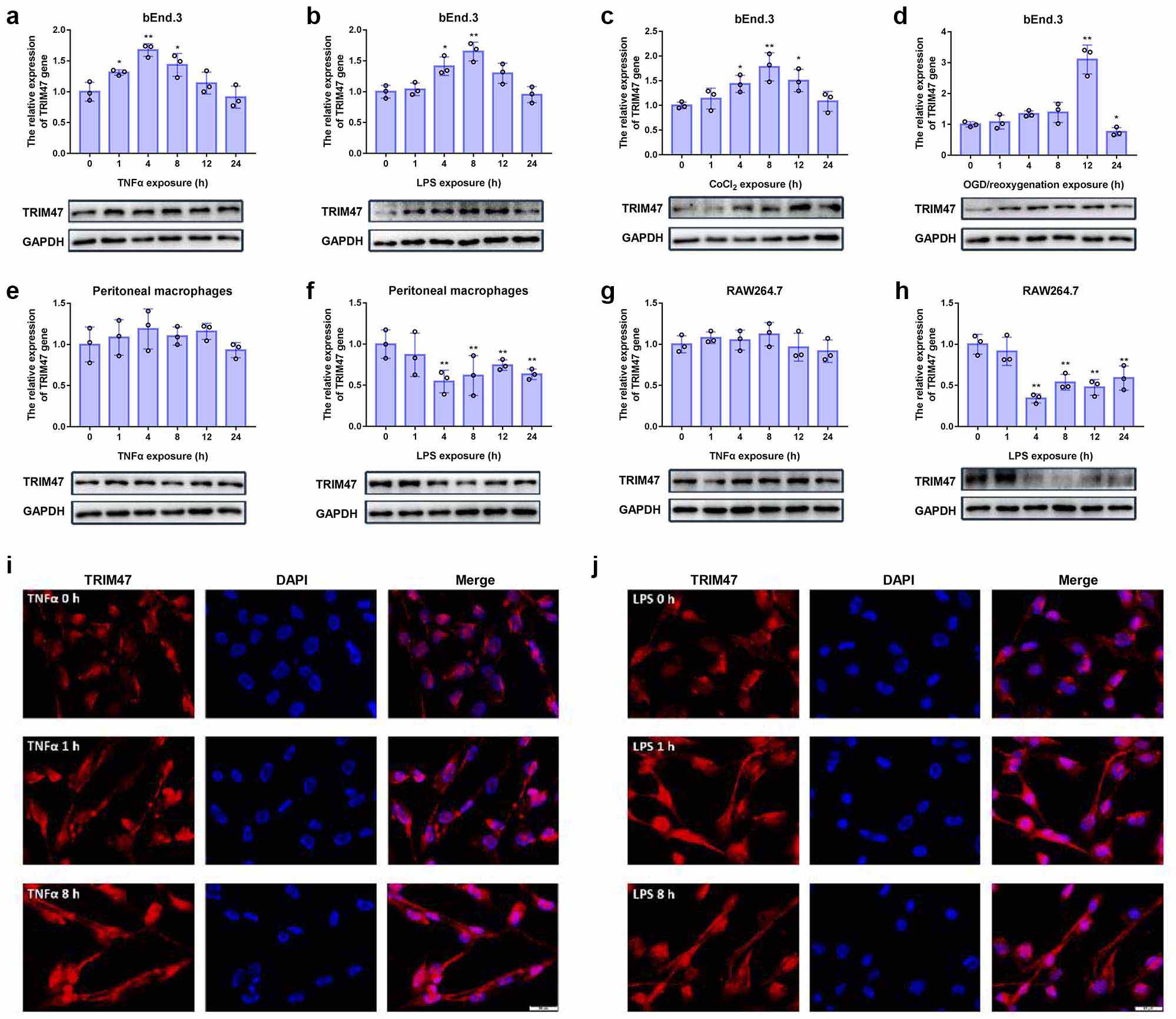


Figure. S2. The expression profile of TRIM47 after stimulation. The expression of TRIM47 was determined by real-time PCR and western blot respectively. The mouse brain microvascular endothelial cells bEnd.3 were challenged with (a) TNFα, (b) LPS, and hypoxia induced by (c) CoCl2 and (d) oxygen glucose deprivation/reoxygenation (OGD/R). The peritoneal macrophages were exposed to (e) TNFα and (f) LPS, and RAW264.7 macrophages were exposed to (g) TNFα and (h) LPS (n = 3, one-way ANOVA, *p < 0.05, **p < 0.01 compared with the 0 h group). The expression of TRIM47 in hCMEC/D3 cells was detected by immunocytochemistry after (i) TNFα and (j) LPS stimulation (n = 3).


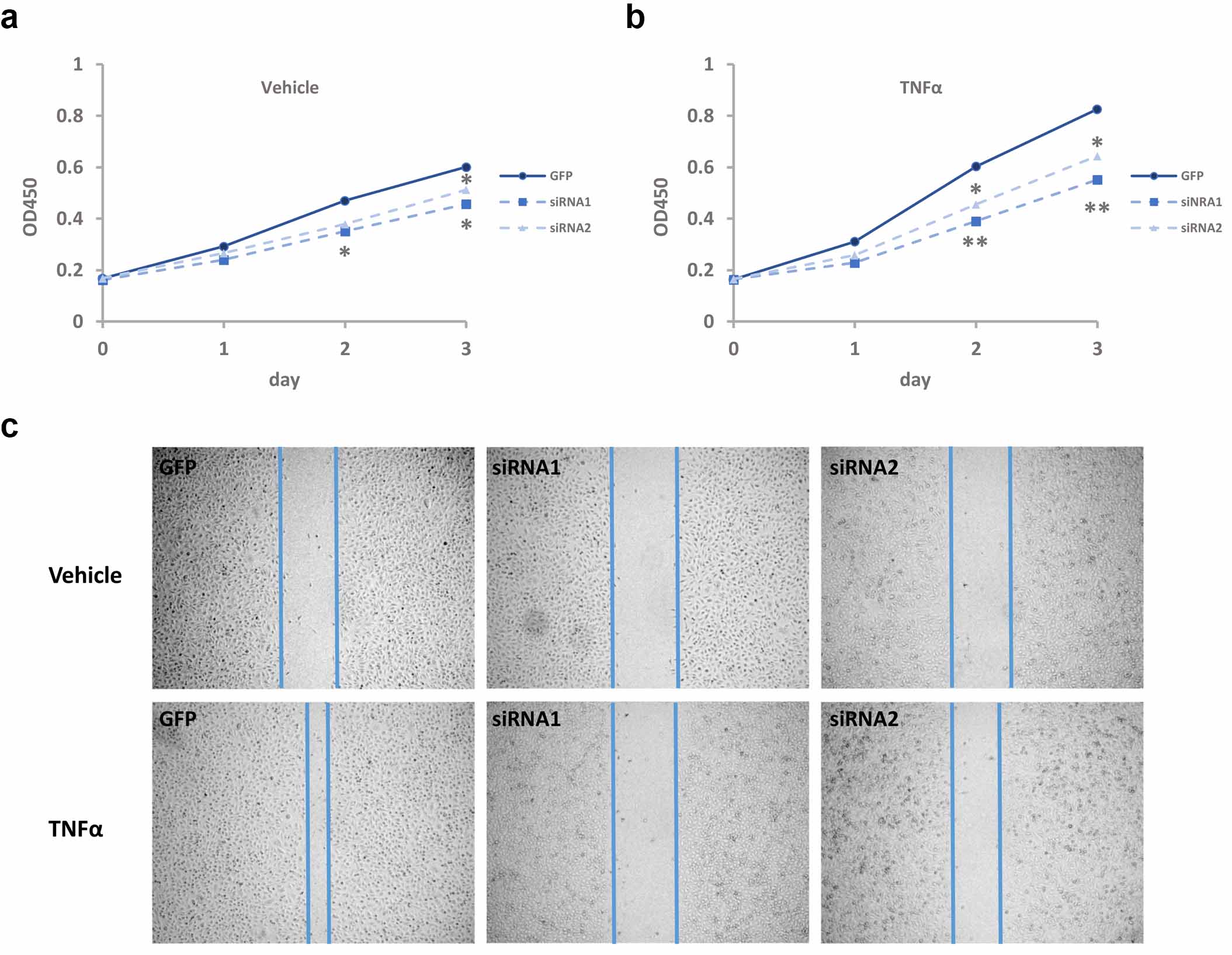


Figure. S3. Knockdown of TRIM47 inhibits cell proliferation and migration induced by TNFα. The proliferation of HUVECs were determined by CCK8 method (A) in the absence and (B) in the absence of TNFα (n = 3). (C) Representative images of cell migration detected by scratch (n = 3).


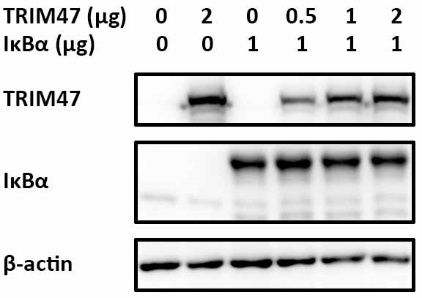


Figure. S4. TRIM47 does not induce IκBα degradation. The TRIM47 and different concentrations of IκBα vectors were co-transfected into HEK293 cells for 24 h. The protein levels of TRIM47 and IκBα were determined by western blot (n = 3).

Table S1. Viscera index in WT and TRIM47 knockout mice (n = 6)

|  | **WT** | **TRIM47 KO** |
| --- | --- | --- |
| Heart | 0.585±0.021 | 0.566±0.010 |
| Liver | 6.814±0.383 | 7.324±0.372 |
| Spleen | 0.330±0.060 | 0.277±0.016** |
| Lung | 0.805±0.020 | 0.859±0.027 |
| Kidney | 0.837±0.032 | 0.822±0.030 |
| Brain | 1.320±0.020 | 1.388±0.052 |

** P <0.01 vs WT.

Table S2. Primers used in real-time PCR reactions

| **Gene** | **Forward primer (5’→3’)** | **Reverse primer (5’→3’)** |
| --- | --- | --- |
| **HumTRIM47** | GAGGGTGCTGTGTCCTATCAACT | CGATAATCTCCACCTCCCAGTAGT |
| **HumVCAM-1** | AGGAAGGCAGTTCTGTGAATATGAC | CCTGCTCCACAGGATTTTCG |
| **HumICAM-1** | CGTTGCCTAAAAAGGAGTTGCT | GCTATCTTCTTGCACATTGCTCAGT |
| **HumE-selectin** | CTGGACTCTCCCTCCTGACATT | ACAAATTTCTTTGCTTTCCGTAAGC |
| **HumMCP-1** | CGCCTCCAGCATGAAAGTCT | GGAATGAAGGTGGCTGCTATG |
| **HumTNFα** | TCTTCTCGAACCCCGAGTGA | GGCCCGGCGGTTCA |
| **HumIL-1β** | CCACAGACCTTCCAGGAGAATG | ATCCCATGTGTCGAAGAAGATAGG |
| **HumIL-6** | TCCAGGAGCCCAGCTATGAA | GAGCAGCCCCAGGGAGAA |
| **HumIL-8** | GCCAAGGAGTGCTAAAGAACTTAGA | TGGTCCACTCTCAATCACTCTCA |
| **HumGAPDH** | CAGGGCTGCTTTTAACTCTGGT | GATTTTGGAGGGATCTCGCT |
| **MusTRIM47** | GGCCCCCAGGGATTACTTC | CCAAAAAGCTGCAGGAACTTG |
| **MusVCAM-1** | GACTCCATGGCCCTCACTTG | GCGTTTAGTGGGCTGTCTATCTG |
| **MusICAM-1** | CACCCCGCAGGTCCAAT | CAGAGCGGCAGAGCAAAAG |
| **MusE-selectin** | GGGACCCAACTGTGAGCAA | ACGGGTGGGAGCAGTTCAG |
| **MusMCP-1** | CTCTCTCTTCCTCCACCACCAT | AGCCGGCAACTGTGAACAG |
| **MusTNFα** | ATCCGCGACGTGGAACTG | ACCGCCTGGAGTTCTGGAA |
| **MusIL-1β** | CTACAGGCTCCGAGATGAACAAC | TCCATTGAGGTGGAGAGCTTTC |
| **MusIL-6** | GTGCAATGGCAATTCTGATTGT | GGTAGCATCCATCATTTCTTTGTATCT |
| **MusGAPDH** | ACATGGCCTCCAAGGAGTAAGAA | GGGATAGGGCCTCTCTTGCT |
